# Highly Conservative Pattern of Sex Chromosome Synapsis and Recombination in Neognathae Birds

**DOI:** 10.3390/genes12091358

**Published:** 2021-08-29

**Authors:** Anna Torgasheva, Lyubov Malinovskaya, Kira S. Zadesenets, Anastasia Slobodchikova, Elena Shnaider, Nikolai Rubtsov, Pavel Borodin

**Affiliations:** 1 Institute of Cytology and Genetics, Russian Academy of Sciences, Siberian Branch, 630090 Novosibirsk, Russia; torgasheva@bionet.nsc.ru (A.T.); Malinovskaya@g.nsu.ru (L.M.); kira_z@bionet.nsc.ru (K.S.Z.); A.Slobodchikova@G.NSU.RU (A.S.); rubt@bionet.nsc.ru (N.R.); 2 Department of Cytology and Genetics, Novosibirsk State University, 630090 Novosibirsk, Russia; 3 Bird of Prey Rehabilitation Centre, 630090 Novosibirsk, Russia; equ001@gmail.com

**Keywords:** avian sex chromosomes, recombination nodules, synaptonemal complex, MLH1, SYCP3, crossing over

## Abstract

We analyzed the synapsis and recombination between Z and W chromosomes in the oocytes of nine neognath species: domestic chicken *Gallus gallus domesticus*, grey goose *Anser anser*, black tern *Chlidonias niger*, common tern *Sterna hirundo*, pale martin *Riparia diluta*, barn swallow *Hirundo rustica*, European pied flycatcher *Ficedula hypoleuca,* great tit *Parus major* and white wagtail *Motacilla alba* using immunolocalization of SYCP3, the main protein of the lateral elements of the synaptonemal complex, and MLH1, the mismatch repair protein marking mature recombination nodules. In all species examined, homologous synapsis occurs in a short region of variable size at the ends of Z and W chromosomes, where a single recombination nodule is located. The remaining parts of the sex chromosomes undergo synaptic adjustment and synapse non-homologously. In 25% of ZW bivalents of white wagtail, synapsis and recombination also occur at the secondary pairing region, which probably resulted from autosome−sex chromosome translocation. Using FISH with a paint probe specific to the germline-restricted chromosome (GRC) of the pale martin on the oocytes of the pale martin, barn swallow and great tit, we showed that both maternally inherited songbird chromosomes (GRC and W) share common sequences.

## 1. Introduction

Comparative genomic analysis indicates that the avian sex chromosomes with ZW heterogamety in females evolved from a pair of autosomes. Regional suppression of recombination between the Z and W chromosomes led to their differentiation [1].

Avian orders differ in the degree of sex chromosome differentiation [2,3]. Among paleognaths, ratites show low differentiation between Z and W. Their sex chromosomes synapse and recombine along 80% of the W long arm (Wq) length [4,5]. Tinamiformes, another order of paleognaths, vary in the length of PAR from 65% of Wq in *Rhynchotus rufescens,* 25% in *Eudromia elegans* and *Nothura maculosa*, to just the very end of Wq in *Crypturellus tataupa* [2]. The recombination suppression in paleognaths is determined by the fixation of different inversions in Z chromosomes in ratites and both in Z and W chromosomes in Tinamiformes [3].

All neognaths examined so far have a very short PAR located near to the telomere of the highly heterochromatic W chromosome. Recombination is suppressed throughout most regions of the sex chromosomes. The genomic analysis made it possible to identify four strata along their sex chromosomes [6]. Adjacent to the S0 stratum, common for both Neognathae and Palaeognathae species, all neognaths share S1 stratum, which was formed about 71 to 119 MYA, before the inferred split between Neoaves–Galloanserae. A younger Neognathae stratum S2 was possibly formed independently in different lineages: 68 (18 to 127) MYA in duck, 69 (66 to 72) MYA in Neoaves [3] and 34 to 54 MYA in chicken [7]. The latest stratum S3 was formed after the divergence between the songbirds and other passerine species. Apparently, it emerged between 45 and 53 MYA at different time points in different lineages probably due to the recent burst of CR1-E1 retrotransposon subfamily elements [8].

The independent formation of strata in different bird lineages, as evidenced by the scaling of the Z/W divergence level in the noncoding regions, indicates that PAR, identified by the highest pairwise-alignment identities between Z/W, may vary in size in different species [3]. However, the extent of the recombining region between the Z and W chromosomes in most bird species remains unknown.

There were several cytological examinations of neognath ZW chromosome pairing and recombination using electron and light microscopic visualization of the synaptonemal complex (SC) and recombination nodules [4,9,10,11,12,13,14,15]. These studies revealed extreme equalization of the heteromorphic sex chromosome axes during meiotic prophase: shortening of Z and elongation of W. They also demonstrated that in all neognaths examined recombination occurred in the very short segment located at the end of W short arm (Wp). 

In this paper, we analyzed the synapsis and recombination between Z and W chromosomes in the oocytes of nine avian species. To trace chromosome synapsis and recombination we used immunostaining with antibodies to SYCP3, the main protein of the lateral elements of the SC, and MLH1, the mismatch repair protein marking mature recombination nodules. We used two sets of data. The first set comprises domestic chicken *Gallus gallus domesticus*, as the reference species and four songbird species that we describe here for the first time: pale martin *Riparia diluta*, barn swallow *Hirundo rustica*, European pied flycatcher *Ficedula hypoleuca* and the great tit *Parus major*. The second set comprises four bird species that have been examined and described in our previous papers: grey goose *Anser anser* [13], black tern *Chlidonias niger*, common tern *Sterna hirundo* [15] and white wagtail *Motacilla alba* [14]. For this set of species, we reanalyzed de novo all images taken earlier and measured them following the same algorithm as in the first set of species. The choice of species was restricted by the narrow time window of availability of the material for the cytological analysis: ovaries of the newly hatched birds. The choice was also restricted by our locality because the chromosome spreads had to be prepared in the laboratory (but not in the field) immediately after slaughter. We focused on the analysis of the songbirds, the most speciose avian order. We also studied two species of terns representing Charadriiformes, another diverse order of birds. We compared these data with the published data on other avian species [2,12,16,17].

The order Passeriformes is marked by an occurrence and wide spreading of the germline-restricted chromosome (GRC). The GRC is present in the germ line, but not in the somatic cells of all songbird species examined so far [18]. The GRC is usually ejected from male germ cells during meiotic divisions and transmitted to the progeny predominantly via females [19,20,21,22]. It contains unique and multiply repeated sequences paralogous to the coding and noncoding regions of the basic genome and interspersed repetitive elements [18,23]. Many GRC-linked genes are found to be expressed and translated in zebra finch female and male germ cells [23]. The relation between GRC and W—two maternally inherited chromosomes—is of special interest [24]. Using FISH with a GRC specific probe, we tried to estimate whether the GRC of the pale martin and W chromosomes of pale martin, barn swallow and great tit share common sequences.

## 2. Materials and Methods

### 2.1. Specimens

Nesting females on days 3–6 after hatching were collected from the nests near Novosibirsk or (in the case of domestic chicken) purchased from the breeders.

Handling and euthanasia of the birds followed the protocols approved by the Animal Care and Use Committee of the Institute of Cytology and Genetics SD RAS (protocols #45/2 of 10 January 2019 and 85 of 15 June 2021). Experiments described in this manuscript were carried out in accordance with the approved national guidelines for the care and use of animals.

### 2.2. SC Spreading and Immunostaining

Chromosome spreads for SC analysis were prepared by the drying down method [25]. Ovaries were quickly washed in phosphate-buffered saline (PBS) and then placed in a hypotonic extraction buffer containing 30 mM Tris, 50 mM sucrose, 17 mM trisodium citrate dihydrate, 5 mM EDTA, pH 8.2, for 30–60 min. The ovaries were then dissected and suspended in 40 μL of 0.1 M sucrose, pH 8.2, on the glass surface. The suspension was dropped onto glass slides moistened with a fresh solution of 1% paraformaldehyde, pH 9.2. The preparations were fixed for 2 h in a humid chamber, then washed twice for 2 min in 0.4% Photoflo and dried in air at room temperature.

Immunostaining was performed according to the protocol of Anderson et al. [26] using rabbit polyclonal anti-SYCP3 (1:500; Abcam, Cambridge, UK), mouse monoclonal anti-MLH1 (1:50; Abcam, Cambridge, UK), and human anticentromere (ACA) (1:100; Antibodies Inc., Davis, CA, USA) primary antibodies. The secondary antibodies used were Cy3-conjugated goat anti-rabbit (1:500; Jackson ImmunoResearch, West Grove, PA, USA), FITC-conjugated goat anti-mouse (1:50; Jackson ImmunoResearch), and AMCA-conjugated donkey anti-human (1:100; Jackson ImmunoResearch). Antibodies were diluted in PBT (3% bovine serum albumin and 0.05% Tween 20 in phosphate-buffered saline). A solution of 10% PBT was used for blocking. Primary antibody incubations were performed overnight in a humid chamber at 37 °C; and secondary antibody incubations, for 1 h at 37 °C. Slides were mounted in Vectashield antifade mounting medium (Vector Laboratories, Burlingame, CA, USA) to reduce fluorescence fading.

### 2.3. Generation of the Microdissected DNA Probe and FISH

DNA probe of pale martin GRC was prepared as described earlier [18]. The cell suspension was prepared from testicular cells of adult males treated with hypotonic solution (0.88% KCl) at 37 °C for 3 h and then with Carnoy’s solution (methanol: glacial acetic acid, 3:1). It was dropped onto clean, cold, wet coverslips, dried, and stained with 0.1% Giemsa solution (Sigma) for 3–5 min at room temperature. Fifteen copies of GRC contained in round Giemsa-positive bodies located near spermatids were microdissected with a glass needle. DNA was extracted from the microdissected material and amplified with the GenomePlex Single Cell Whole Genome Amplification Kit (WGA4) (Sigma-Aldrich, St. Louis, MO, USA) [27]. The PCR product was labeled with Flu-dUTP (Genetyx, Novosibirsk, Russia) in additional PCR cycles (Sileks, Moscow, Russia). FISH experiments on the SC spreads were performed according to a standard protocol with salmon sperm DNA (Ambion, Austin, TX, USA) as a DNA carrier [28].

### 2.4. Microscopic Analysis

Images of SC spreads after immunostaining were captured using a CCD-camera installed on the Axioplan 2 compound microscope (Carl Zeiss, Oberkochen, Germany) equipped with filter cubes #49, #10, and #15 (Carl Zeiss, Oberkochen, Germany) using ISIS4 (METASystems GmbH, Altlußheim, Germany) at the Center for Microscopic Analysis of Biological Objects of SD RAS (Novosibirsk, Russia). Corel PaintShop Pro X6 (Corel, Ottawa, ON, Canada) was used for a correction of image brightness and contrast.

### 2.5. Chromosome Measurements and Generation of Recombination Maps of GRCs

Only cells containing complete sets of chromosomes were analyzed. We measured length and centromere indices of Z and W chromosomes in pachytene oocytes with most autosomes completely paired. Two types of sex chromosome synaptic configuration were measured. First, asynapsed Z and W chromosomes were identified as univalents of different lengths in early pachytene. If no cells with asynapsed sex chromosomes were observed, we measured axial elements of partially paired ZW bivalent. In this case, we only used cells where synapsis did not extend beyond the centromere of the W chromosome. Second, completely paired ZW bivalents, which formed in mid-late pachytene, were identified by misaligned centromeres. Centromeres were identified by ACA foci. MLH1 signals were only scored if they were localized on SCs. The distance from MLH1 to telomere was measured in completely paired ZW bivalents. The length of the SC was measured in micrometers and the positions of MLH1 foci in relation to the centromere were recorded using MicroMeasure 3.3 [29]. STATISTICA 6.0 software package (StatSoft, Tulsa, OK, USA) was used for calculating means and standard deviations. The results were expressed as mean ± SD.

## 3. Results

### 3.1. Common Features of ZW Synapsis

The meiotic behavior of ZW chromosomes of the bird species studied and re-examined within the framework of this project was rather similar to that described in other Neognathae birds examined to date [2,9,11,12,16,17,30].

**Figure 1:**
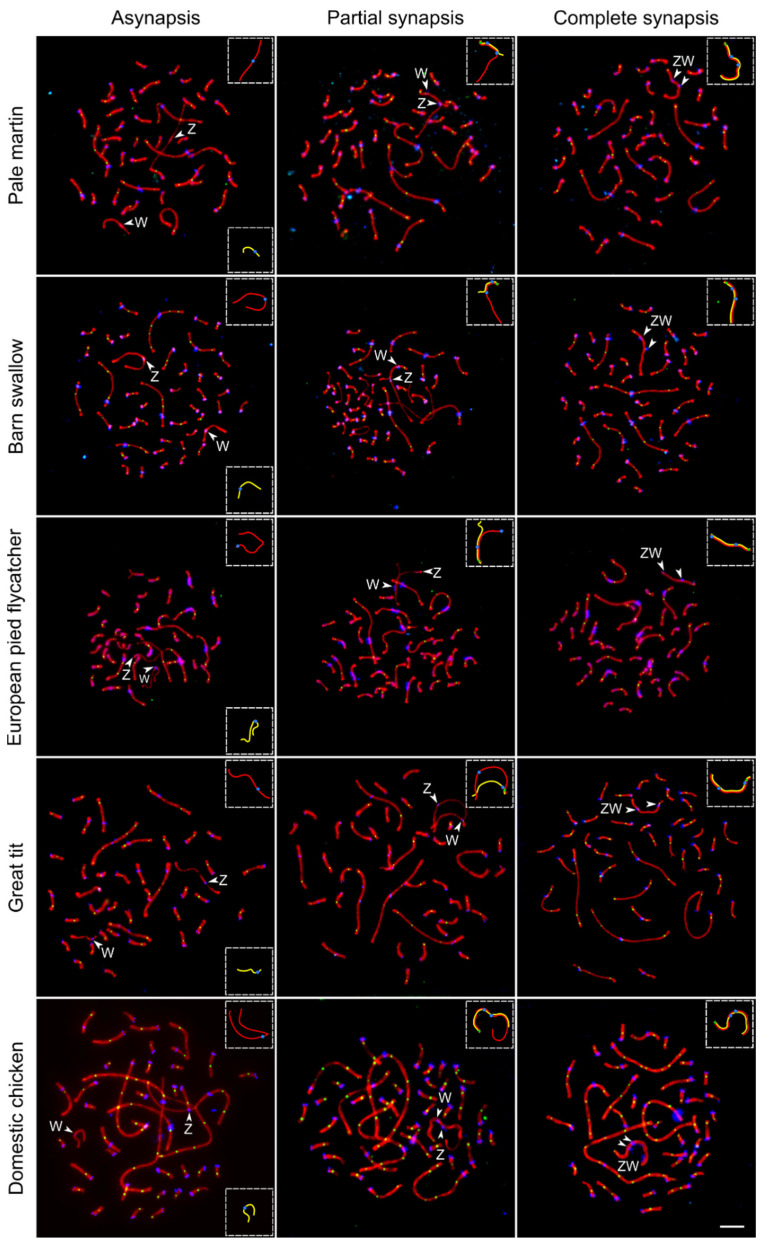
Synaptic configurations of the ZW SCs during pachytene in five bird species visualized by anti-SYCP3 (red), anti-MLH1 (green) and anticentromere (blue) antibodies. Arrowheads indicate centromeres of Z and W chromosomes. Inserts show schematic representations of Z (red) and W (yellow) SCs. Bar—5 µm.

Figure 1 shows the sequential stages of Z and W chromosome synapsis in the domestic chicken and four songbird species examined for the first time: pale martin, barn swallow, European pied flycatcher and great tit. Similar images taken from the SC spreads of four other birds species (grey goose, black tern, common tern and white wagtail) have been published earlier [13,14,15]. The pairing of Z and W chromosomes was delayed compared to synapsis of autosomes. A large fraction of oocytes contained completely or partially unpaired Z and W chromosomes while all autosomes were completely synapsed (Figure 1, left and mid columns). The length of unpaired W univalent varied from 35% of unpaired Z univalent in grey goose to 70% in black tern (Table 1). Sex chromosomes started to pair at the ends of W and Z chromosomes (Figure 1, middle column). Synapsis was initiated at Wp in all species examined and at Zp in the most bird species studied, except great tit and European pied flycatcher. In the latter species, the pairing region was located at Zq, probably due to the shift of the centromere.

**Figure 2:**
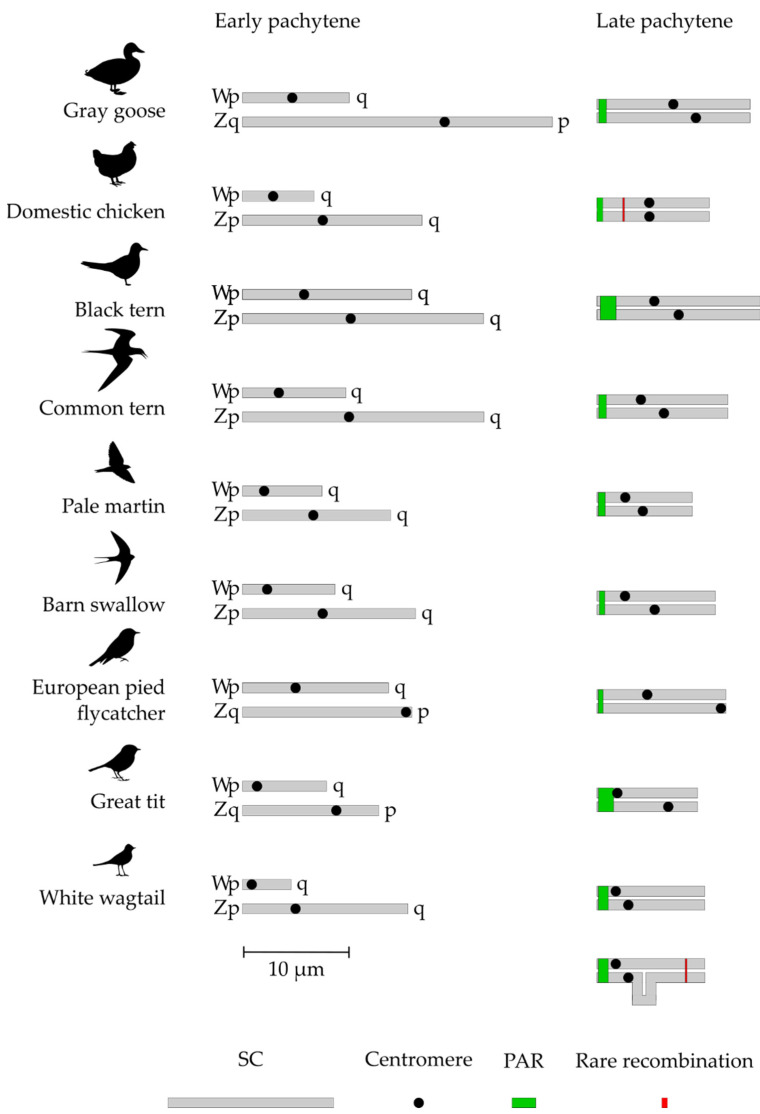
Schematic representation of Z and W SCs before and after synaptic adjustment.

After synapsis was established, it expanded to the differentiated parts of the sex chromosomes. The expansion was accompanied by synaptic adjustment and axis equalization (shortening of Z-axis and elongation of W-axis). It was completed by a formation of heteromorphic ZW SCs marked by two distinct misaligned centromere signals. (Figure 1, right column). Figure 2 depicts the sizes of Z and W chromosomes and the positions of their centromeres before and after the equalization. The relative length of completely paired ZW bivalent was about 5% of the total SC length per cell and did not differ substantially between the species (Table 1).

**Figure 3:**
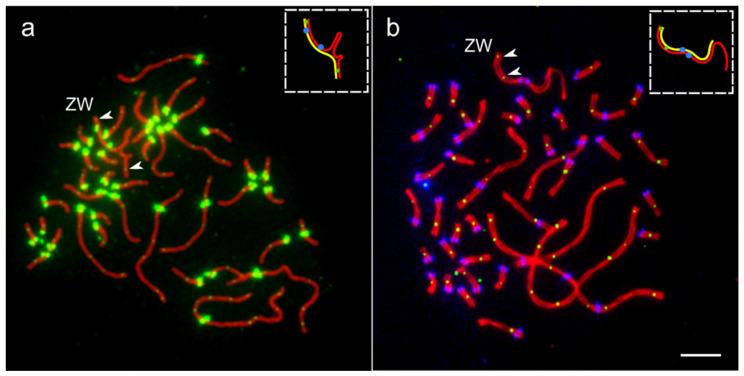
Two exceptional oocytes with two MLH1 signals at ZW bivalents. (**a**) Pachytene oocyte of the white wagtail immunostained with antibodies against SYCP3 (red), MLH1 (small green dots) and centromeres (large green dots). (**b**) Pachytene oocyte of domestic chicken immunostained with antibodies against SYCP3 (red), MLH1 (small green dots) and centromeres (large blue dots). Arrowheads indicate MLH1 signals at ZW bivalents. Inserts show schematic representations of Z (red) and W (yellow) SCs. Bar—5 µm.

As we described earlier [14], about 25% of oocytes of the white wagtail contained ZW bivalent independently synapsed at both ends of the sex chromosomes. Figure 3a shows one of such synaptic configurations.

### 3.2. MLH1 Foci Distribution along ZW Bivalents

Most ZW bivalents in all species examined contained a single MLH1 focus. The average absolute and relative distances of MLH1 foci from the telomere in the completely paired ZW SCs are shown in Table 1. We used the limits of MLH1 foci distribution to outline the location and size of the region where recombination occurs (PAR sensu stricto). The size of this region varied from 5% of the completely paired ZW bivalent in the domestic chicken and grey goose to almost 14% of that in the great tit (Table 1). In the great tit and the white wagtail, PAR included almost the whole Wp (Figure 2). 

In the white wagtail and the domestic chicken, we found exceptional oocytes with two MLH1 signals at ZW bivalents. 

When we reanalyzed the images of the immunostained cells of white wagtail oocytes, in one of 35 oocytes with two pairing regions, we detected one MLH1 focus at the primary pairing region Zp-Wp and another one in the secondary pairing region Zq-Wq (Figure 3a). It was located at 0.7 µm from the telomere (5% of the length of Z chromosome).

In one oocyte of the domestic chicken, we detected two clear MLH1 foci at the Zp-Wp pairing region (Figure 3b). The first was located close to the telomere, while the second was located at 2.5 µm from the telomere (21% of the length of Z chromosome), far beyond the most distant single MLH1 focus detected in other chicken oocytes.

### 3.3. FISH of Whole-Chromosome Probe of the Pale Martin GRC with Pachytene Chromosomes of Three Songbird Species 

Using the whole-chromosome probe of the pale martin GRC, we carried out FISH on the pachytene oocytes of the pale martin, barn swallow and great tit. 

**Figure 4:**
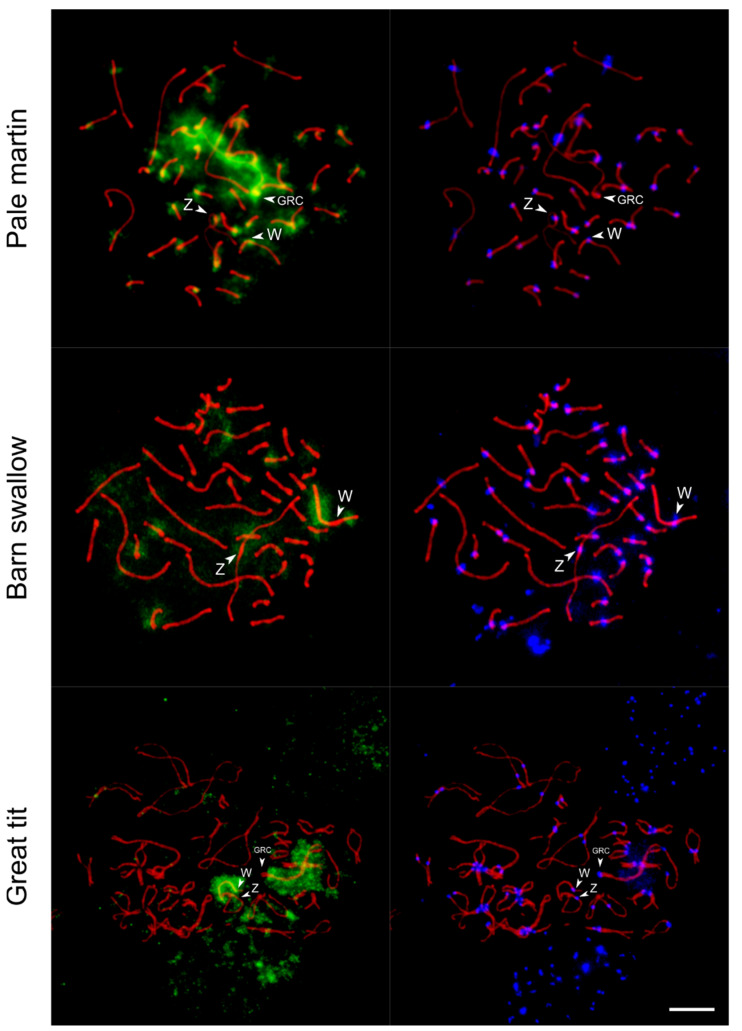
FISH with pale martin GRC-specific probe (green) on pachytene oocytes of pale martin and barn swallow and diplotene oocyte of great tit immunostained with antibodies to SYCP3 (red) and centromere proteins (blue). Arrowheads show centromeres of Z, W and GRC chromosomes. In barn swallow, GRC is one of the microchromosomes. Bar—5 µm.

In all these experiments, we detected hybridization signals at the W chromosome, although at its different regions (Figure 4). In the pale martin, FISH with the GRC-specific probe intensely painted GRC and pericentromeric regions of most chromosomes, including Z and W chromosomes. There were also less prominent signals at some regions on autosomes and W chromosome arms except for the interstitial region of Wq.

Consistent with the previous study [18], the signal on GRC in the interspecies FISH experiments was much less intense. In the absence of a highly intense signal on GRC, the labeling on the W chromosome of the barn swallow and great tit was more prominent. In the barn swallow, which carries micro-GRC [18], FISH with the GRC-specific probe of the pale martin painted the interstitial regions of Wp and Wq arms. Signals of lower intensity were also observed at regions of several autosomes and at the pericentromeric region of the Z chromosome. In the great tit, a specific FISH signal was observed along almost the whole W chromosome except the end of the Wp arm. Less intense FISH signals were also revealed in some regions of several autosomes and the Z chromosome. Notably, in all three species FISH with the GRC probe did not label PAR in Z and W chromosomes.

Despite the presence of shared sequences in GRC on the one hand, and in W and some autosome regions on the other hand, we did not detect pairing between GRC and sex chromosomes or autosomes even at early pachytene when some autosomes show ectopic contacts.

## 4. Discussion

The analysis of the synapsis and recombination of Z and W chromosomes in nine species of neognath birds confirmed the conclusions about the meiotic behavior of the sex chromosomes in this infraclass drawn earlier [12]. Obligatory homologous synapsis and recombination occur in PAR located at the ends of Z and W chromosomes and restricted by one crossover. The remaining parts of the sex chromosomes undergo synaptic adjustment and synapse non-homologously. Despite the complex evolutionary stratification of the avian sex chromosomes, different in different lineages [3,6,7,8], the size of PAR in the species examined varies within rather narrow limits (Figure 2). 

The only exception is the ZW bivalent of the white wagtail, which often show synapsis at its both ends [14]. The two pairing regions are spaced by regions of asynapsis or self-synapsed hairpins. This indicates that synapsis in both the pairing regions is initiated independently. The initiation of the synapsis requires extended homology for the invasion of single-strand DNA and formation of the stable recombination intermediates. These intermediates can be resolved via noncrossover or crossover pathways [31]. Although the occurrence of MLH1 focus in the secondary pairing region can be an artifact, it is expected and it might be considered as a confirmation that recombination in this region is possible, although in a low frequency. In its appearance and the frequency of recombination events, the secondary PAR of the white wagtail resembles the human secondary PAR. SC analysis shows that from 25 to 70% of human XY are paired at both ends [32]. Genetic analysis made it possible to detect recombination in the human PAR2 in approximately 2% of male meioses [33], although recombination nodules have never been observed (or reported) in this region [32,34].

The secondary PAR of the white wagtail might have resulted from autosome−sex chromosome transposition. Evidence of such transpositions have been found in birds [35,36,37,38,39]. Close molecular genetic examination of the white wagtail sex chromosomes is necessary to check this hypothesis.

Genomic data indicate that the DNA sequences located in Z and W retain a relatively high level of homology despite many million years of independent evolution [3]. Our finding of the exceptional chicken oocyte with ZW bivalent with one MLH1 signal close to the telomere and another one far beyond the limit of MLH1 distribution in PAR indicates that rare recombination events between these dispersed regions of residual homology are possible. We cannot exclude that the exceptional MLH1 focus at ZW SC of the single chicken oocyte could be an artifact. On the other hand, recombination events in the differentiated regions of the sex chromosomes should be exceptionally rare.

The results of FISH with the DNA probe derived from the pale martin GRC indicate that copies of its sequences might be present in the W chromosome of all three songbird species examined. This finding is probably explained by the accumulation of shared repetitive elements on both the GRC and W chromosome, which are heterochromatic and do not recombine along most of their length. Cytogenetic and genomic studies demonstrate that GRC is a highly dynamic chromosome, containing multiple copies of genes located at almost all autosomes and Z chromosome and probably characterized by up to 1.5 times the density of interspersed repetitive elements than the genome average [18,23,40,41]. A recent study revealed that the W chromosome, unlike autosomes, is enriched with transposable elements and contains the majority of genome full-length endogenous retroviruses (ERV) exhibiting female-specific expression [42]. Asalone suggested that ERV-driven retrotransposition within females might be a source of gaining and duplication of genes in GRC [43]. Speculatively, the abundance of active ERVs in the W chromosome might generate DNA traffic to GRC and could lead to the enrichment of GRC with copies of repetitive and probably unique sequences from the W chromosome.

## Figures and Tables

**Table 1:** Synapsis and recombination-related characteristics of Z and W chromosomes in the bird species examined.

Species	Domestic Chicken	Grey Goose	Black Tern	Common Tern	Pale Martin	Barn Swallow	European Pied Flycatcher	Great Tit	White Wagtail
Number of birds	3	3	1	2	3	3	2	4	2
Pairing arms	Zp-Wp	Zq-Wp	Zp-Wp	Zp-Wp	Zp-Wp	Zp-Wp	Zq-Wp	Zq-Wp	Zp-Wp and Zq-Wq
Complete synapsis									
N *	57	50	89	150	61	128	80	152	57
Absolute length of ZW, µm	10.7 ± 3.3	14.6 ± 4.0	15.6 ± 3.3	12.5 ± 2.1	9.1 ± 2.5	11.3 ± 1.8	12.3 ± 3.4	9.1 ± 1.6	10.3 ± 1.7
Relative length of ZW **, %	4.7	4.9	5.1	5	5.1	5.7	5.4	4.6	5.3
CI of Z	0.47 ± 0.03	0.36 ± 0.06	0.51 ± 0.06	0.52 ± 0.04	0.48 ± 0.03	0.49 ± 0.05	0.02 ± 0.03	0.29 ± 0.05	0.29 ± 0.05
CI of W	0.50 ± 0.06	0.50 ± 0.07	0.35 ± 0.04	0.34 ± 0.04	0.30 ± 0.04	0.24 ± 0.04	0.39 ± 0.03	0.16 ± 0.04	0.17 ± 0.04
Asynapsis (A) or partial synapsis (P)									
Synapsis	A/P	P	P	A	A	A	A	A	A
N	25	6	28	19	43	59	16	28	15
Length of Z, µm	17.1 ± 4.1	29.5 ± 6.8	23.0 ± 5.7	23.0 ± 4.6	14.1 ± 2.1	16.5 ± 4.4	16.7 ± 3.8	11.9 ± 3.6	15.8 ± 2.3
CI of Z	0.45 ± 0.04	0.35 ± 0.04	0.46 ± 0.07	0.44 ± 0.05	0.48 ± 0.01	0.47 ± 0.02	0.03 ± 0.02	0.31 ± 0.05	0.32 ± 0.02
Length of W, µm	8.68 ± 1.7	10.2 ± 3.1	16.1 ± 2.9	9.9 ± 2.1	7.6 ± 1.0	8.8 ± 1.9	11.3 ± 1.8	7.0 ± 1.41	4.7 ± 0.8
CI of W	0.44 ± 0.05	0.47 ± 0.07	0.36 ± 0.07	0.36 ± 0.04	0.28 ± 0.04	0.28 ± 0.05	0.41 ± 0.04	0.16 ± 0.04	0.19 ± 0.05
Distance to MLH1 focus from the telomere in completely synapsed ZW									
N	57	50	89	150	61	128	80	152	57
Average distance, µm	0.12±0.18	0.45 ± 0.17	0.79 ± 0.24	0.62 ± 0.17	0.45 ± 0.14	0.53 ± 0.13	0.37 ± 0.12	0.82 ± 0.26	0.37 ± 16
Average distance ***, %	1.0 ± 1.5	3.4 ± 1.3	5.2 ± 1.6	5.1 ± 1.5	5.0 ± 1.5	4.8 ± 1.2	3.3 ± 1.3	9.0 ± 2.9	3.6 ± 1.6
Min-max distance, µm	0–0.58	0.10–0.83	0.31–1.85	0.18–0.97	0.16–0.89	0.27–0.87	0.15–0.68	0.20–1.70	0.17–1.24
Min-max distance, %	0–4.7	0.9–5.7	1.4–10.4	1.2–8.4	1.5–8.3	2.5–8.4	1.0–6.6	2.0–15.6	1.4–12.5
% of ZW occupied by MLH1 foci	4.7	4.8	9	7.2	6.8	5.9	5.6	13.6	11.1

* N—number of cells examined, ** Relative length of ZW—ratio ZW SC length to total SC length, *** Relative distance—% of paired ZW.

## Data Availability

Publicly available datasets were analyzed in this study. These data can be found here: https://meiosislab.com/projects/chromosomes/zwbirds.xlsx accessed on 27 August 2021.
